# Reassessing Breast Cancer-Associated Fibroblasts (CAFs) Interactions with Other Stromal Components and Clinico-Pathologic Parameters by Using Immunohistochemistry and Digital Image Analysis (DIA)

**DOI:** 10.3390/cancers15153823

**Published:** 2023-07-27

**Authors:** Alina Cristina Barb, Mihaela Pasca Fenesan, Marilena Pirtea, Mădălin-Marius Margan, Larisa Tomescu, Emil Ceban, Anca Maria Cimpean, Eugen Melnic

**Affiliations:** 1 Department of Microscopic Morphology/Histology, Victor Babes University of Medicine and Pharmacy, 300041 Timisoara, Romania; toma.alina@umft.ro (A.C.B.); fenesan.mihaela@gmail.com (M.P.F.); pirtea.laurentiu@umft.ro (M.P.); 2 Doctoral School in Medicine, Victor Babes University of Medicine and Pharmacy, 300041 Timisoara, Romania; tomescu.larisa@umft.ro; 3 Department of Clinical Oncology, OncoHelp Hospital, 300239 Timisoara, Romania; 4 Department of Functional Sciences/Discipline of Public Health, Victor Babes University of Medicine and Pharmacy, 300041 Timisoara, Romania; marganmm@gmail.com; 5 Department of Obstetrics and Gynecology, “Victor Babes” University of Medicine and Pharmacy Timisoara, 300041 Timisoara, Romania; 6 Department of Urology and Surgical Nephrology, Nicolae Testemitanu State University of Medicine and Pharmacy, 2004 Chisinau, Moldova; emil.ceban@usmf.md; 7 Laboratory of Andrology, Functional Urology and Sexual Medicine, Nicolae Testemitanu State University of Medicine and Pharmacy, 2004 Chisinau, Moldova; 8 Center of Expertise for Rare Vascular Disease in Children, Emergency Hospital for Children Louis Turcanu, 300011 Timisoara, Romania; 9 Department of Pathology, Nicolae Testemitanu State University of Medicine and Pharmacy, 2004 Chisinau, Moldova; eugen.melnic@usmf.md

**Keywords:** cancer-associated fibroblasts (CAFs), CD34_CAFs, αSMA_CAFs, immunohistochemistry (IHC), digital image analysis (DIA), breast cancer (BC), tumor stroma

## Abstract

**Simple Summary:**

Breast cancer (BC) is primarily classified by immunohistochemistry (IHC) and digital image analysis (DIA) based on distinct immunophenotypes of malignant epithelial cells. When antiangiogenic and immunomodulatory therapies are used for BC, the stromal components such as tumor vessels and immune cells have received more attention. Cancer-associated fibroblasts (CAFs) are extensively investigated, but measuring them in immunostained tissues and determining how they relate to clinical and pathological criteria is still difficult in BC. Our earlier research using CD34 and α-smooth muscle actin (αSMA) immunohistochemistry showed a decrease of CD34-positive CAFs and an increase of αSMA-positive CAFs inside the tumor stroma. This early basic microscopic finding gives us the intention to employ DIA to improve SMA-positive CAF quantification accuracy. Additional DIA processed data correlations to pathologic parameters, survival, invasion, and recurrence indicated substantial differences between BC molecular subtypes, suggesting a unique CD34- and αSMA-positive CAF impact for each subtype.

**Abstract:**

Background: Breast cancer (BC) stroma has CD34- and αSMA-positive cancer-associated fibroblasts (CAFs) differently distributed. During malignant transformation, CD34-positive fibroblasts decrease while αSMA-positive CAFs increase. The prevalence of αSMA-positive CAFs in BC stroma makes microscopic examination difficult without digital image analysis processing (DIA). DIA was used to compare CD34- and αSMA-positive CAFs among breast cancer molecular subgroups. DIA-derived data were linked to age, survival, tumor stroma vessels, tertiary lymphoid structures (TLS), invasion, and recurrence. Methods: Double immunostaining for CD34 and αSMA showed different CAF distribution patterns in normal and BC tissues. Single CD34 immunohistochemistry on supplemental slides quantified tumor stroma CD34_CAFs. Digital image analysis (DIA) data on CAF density, intensity, stromal score, and H-score were correlated with clinico-pathologic factors. Results: CD34/αSMA CAF proportion was significantly related to age in Luminal A (LA), Luminal B (LB), and HER2 subtypes. CD34_CAF influence on survival, invasion, and recurrence of LA, LB-HER2, and TNBC subtypes was found to be significant. The CD34/αSMA-expressing CAFs exhibited a heterogeneous impact on stromal vasculature and TLS. Conclusion: BC stromal CD34_CAFs/αSMA_CAFs have an impact on survival, invasion, and recurrence differently between BC molecular subtypes. The tumor stroma DIA assessment may have predictive potential to prognosis and long-term follow-up of patients with breast cancer.

## 1. Introduction

Cancer-associated fibroblasts (CAFs) represent a significant constituent of the tumor microenvironment in cases of breast cancer (BC). Prior research has demonstrated that these entities are notably diverse and intimately linked with tumor cells and other stromal constituents, such as tumor stroma vessels or immune cells. However, the precise mechanisms underlying these intricate interactions remain incompletely understood [1,2]. Various CAF markers have been employed to categorize CAFs in breast cancer into distinct subgroups, potentially exerting a unique influence on molecular subtypes of breast cancer. Despite the heterogeneity of markers for cancer-associated fibroblasts (CAFs), there is currently no specific marker that can accurately define each type of CAF. Ongoing efforts are being made to identify additional markers for this purpose [1,3]. Research on the tumor microenvironment’s cancer-associated fibroblasts (CAFs) has extended beyond the stroma of primary cancers. In recent publications, there has been a presentation of data on the heterogeneity of CAFs from the stroma of metastases [4,5]. Vimentin, fibroblast-associated protein (FAP), CD34, and alpha smooth muscle actin (αSMA) are the most frequently utilized markers for evaluating cancer-associated fibroblasts (CAFs). Divergent expression of CD34-positive fibroblasts in breast normal tissue and alpha smooth muscle actin (αSMA) cancer-associated fibroblasts (CAFs) inside breast cancer (BC) stromal compartment is not a new topic in the field [6,7,8,9,10], but its prognostic and therapeutic impact is still controversial [8,11] and incompletely understood [12,13,14,15]. Most of these studies were performed on classical BC histopathology, and few of them assessed CD34 fibroblasts and αSMA_CAFs related to different molecular subtypes. Despite the recently reported complex heterogeneity markers expressed by CAFs [16], CAFs’ prognostic and therapeutic role is extremely limited [16,17]. Most of the studies focused on CD34/αSMA_CAF interplay in BC stroma used manual semiquantitative methods to assess immunohistochemical staining and to classify them as “*present*” or “*absent*”. Also, the stratification of immunostaining intensity is highly influenced by different perceptions of people who assessed BC specimens by conventional microscopic methods. This is a very limited subjective method related to the lack of its ability to count positive cell density (especially for αSMA-positive cells which are present in a high amount inside tumor stroma and, moreover, are closely packed or form networks, thus being impossible to count) [18,19,20,21]. On the other side, the CD34-positive fibroblasts dramatically decrease from normal breast stroma to tumor stroma, and they are wrongly considered to be absent inside stromal compartment surrounding malignant areas [6,18]. This basic interpretation seems to be the main reason why there are scattered correlative data in between CD34_CAFs and prognostic factors in BC. CD34-positive stromal cells are usually named stromal telocytes and are reported to be stromal progenitor cells with a high ability to differentiate into other stromal cells including αSMA-positive myofibroblasts during tissue repair, regeneration, inflammatory processes, or malignancies [22,23,24,25,26,27]. A pathologist who evaluates a BC tissue slide is highly tempted to be focused on tumor cells rather than cellular and vascular stromal components. A routine IHC antibody panel for BC evaluation does not usually include CD34 and αSMA due to the lack of their expression in tumor cells. CD34 may be used as marker for tumor blood vessels when the pathologist needs vascular invasion confirmation [28]. αSMA immunostaining is used during routine histopathologic diagnosis as a marker for myoepithelial cells and is helpful to evaluate controversies related to microinvasion or for papillary and spindle cells lesions [28]. At this time, BC stroma assessment is not included by pathologists in routine diagnostic evaluation of BC patients, and it has no certified predictive or prognostic role in routine microscopic evaluation despite the recent data which mentioned BC tumor stroma as a potential target for innovative therapies [29,30]. Currently, there are no established guidelines on how to process and evaluate BC stroma CD34 and αSMA CAFs, most likely due to the lack of proper tools to accomplish this. The tumor–stroma ratio proved itself to be a predictor and have a prognostic impact on BC based upon the results of retrospective studies performed on human BC specimens [31,32,33]. For tumor–stroma ratio evaluation, there are already well-developed and standardized digital tools for automated assessment [34,35]. The ability of the human eye to evaluate histology slides is limited. Hence, computer-aided quantitative digital image analysis (DIA) offers a more thorough evaluation of many components [36,37,38,39]. However, due to its time-consuming nature and limited accessibility, this technique is not frequently employed. Lumongga et al. [40] assessed vimentin positive tumor stroma fibroblasts by their intensity but not by their density using Image J as a digital image analysis tool, and this assessment was limited to triple negative breast cancer molecular subtype [40].

The investigation of CAFs has been the subject of extensive research. However, the measurement of CAFs in immunostained tissues and the determination of their relationship with clinical and pathological criteria remain challenging in the context of breast cancer. Our previous investigations utilizing CD34 and α-smooth muscle actin (αSMA) immunohistochemistry revealed a reduction in CD34-positive cancer-associated fibroblasts (CAFs) and a rise in αSMA-positive CAFs within the tumor stroma [6]. This preliminary microscopic observation leads us to the aim of this present study to use digital image analysis (DIA) to enhance the precision of quantifying smooth muscle actin (SMA)-positive cancer-associated fibroblasts (CAFs). The analysis of further processed data by DIA could reveal significant variations among the molecular subtypes of breast cancer in terms of pathologic parameters, survival, invasion, and recurrence. Based on all these previous data, we hypothesized that the impact of CD34- and αSMA-positive cancer-associated fibroblasts (CAFs) may be distinct for each subtype and may be accurately enhanced by using DIA associated with immunohistochemistry interpretation.

We propose here the quantification of CD34 and αSMA tumor stromal CAFs by using a stromal score (SS) which combines both intensity and density of stromal positive cells and H-score automatically generated by QuPath digital image analysis software. Density and intensity of positive stromal cells together with SS and H-score will be interpreted related to their impact on age, survival rate, BC molecular subtype, vascular and immune stromal components, invasion, and recurrence.

## 2. Materials and Methods

### 2.1. The Selection of Patients and Ethical Considerations

The present investigation is a part of a larger retrospective study that includes 150 breast cancer formalin-fixed paraffin-embedded (FFPE) specimens obtained from women with ductal invasive carcinoma on histopathology identified between 2015 and 2022 and ranging in age from 32 to 85 years old. Two different pathologists performed a second round of analysis on the FFPE tissues to confirm the diagnosis and select cases for immunohistochemistry. A molecular profile was determined for each individual case using immunohistochemistry markers. These markers included the Estrogen Receptor (ER), the Progesterone Receptor (PR), the Proliferation Index (Ki67), and an evaluation for the Human Epidermal Growth Factor Receptor 2 (HER2). This was necessary to determine the different breast cancer molecular subtypes. For the goal of this investigation, we chose 53 patients who possessed a complete clinical, histopathological, and therapeutic profile that would be helpful for our purposes. Age, menopausal status, breast cancer molecular subtype, tumor grade (G), Nottingham Prognostic Index (NPI), lymphovascular invasion, perineurial invasion, and recurrence were chosen as the clinicopathologic and therapeutic factors for this study. From stromal components, we selected tertiary lymphoid structures (TLS, previously assessed on the same cases) and immature (CD34+/αSMA-) or mature (CD34+/αSMA+) stromal tumor blood vessels to be correlated with stromal CAFs. At the time of the diagnosis, none of the patients had a previous history of metastases that could be confirmed by imaging techniques. Based on a standardized form that had previously been reviewed and approved by the Research Ethics Council of the “Victor Babeş” University of Medicine and Pharmacy in Timișoara (No. 49/28.09.2018), informed permission was obtained from each patient.

### 2.2. Initial Processing, Histology, and Selection of Formalin-Fixed Paraffin-Embedded (FFPE) Specimens for Immunohistochemistry

Briefly, pieces of the tumor were obtained for the initial diagnosis before any treatment was administered using a combination of mastectomy and needle core biopsy. A piece of the tumor and the area surrounding the tumor that was indicative of the whole were chosen. After obtaining breast cancer tissue samples and storing them in buffered formalin for 24 to 48 h, the specimens were then embedded in paraffin according to the conventional method. Each FFPE block was sectioned to a thickness of three micrometers, and the resulting sections were put on glass slides. In order to do a histopathologic examination, one slide was taken from each case and stained with hematoxylin and eosin. Two separate pathologists evaluated the hematoxylin and eosin-stained slides that were pertinent to validate the initial histopathological diagnosis and evaluate the tissue quality. This was important to make the appropriate case selection for immunohistochemistry. The TLS was detected and quantified by the pathologists, and this was accomplished in conjunction with the histopathologic diagnosis. Vimentin immunostaining was utilized as a means of determining the overall tissue quality (clone V9). Cases with a positive vimentin staining in the tumor stroma were regarded suitable for selection and for subsequent immunohistochemical studies.

### 2.3. Immunohistochemistry

Immunohistochemistry (IHC) was carried out on sections that were three micrometers in thickness utilizing an autostainer manufactured by Leica Biosystems (Newcastle upon Tyne, UK). The Novocastra Bond Epitope Retrieval Solution 1 and 2 together were utilized in the process of unmasking (Leica Biosystems, Newcastle Ltd., Newcastle Upon Tyne, UK). Hydrogen peroxide at a concentration of 3% was used to inhibit endogenous peroxidase activity for a period of five minutes. A method using double immunostaining was carried out on some tissue specimens in order to conduct the interplay in between CD34-positive fibroblasts known to be common for normal breast tissue stroma and SMA_CAFs highly present inside the stromal component of BC tissue. A differentiated analysis of mature and immature tumor blood vessels originating from the BC stromal component was also performed and analyzed previously, and data were correlated to CAFs parameters. We used CD34 mouse anti-human monoclonal antibodies (clone, QBEnd 10, Leica Biosystems, Newcastle upon Tyne, UK, 30 min incubation time at room temperature) for the endothelium of tumor blood vessels and fibroblasts from normal and tumor stroma. Additionally, we used alpha smooth muscle actin (SMA) mouse anti-human monoclonal antibodies (clone 1A4, Leica Biosystems, Newcastle upon Tyne, UK, 30 min incubation time at room temperature) for perivascular cells and CAFs from the tumor stroma. The completion of the immunohistochemical method required the use of visualization systems such as the Bond Polymer Refine Detection System DAB and the Bond Polymer Refine Red Detection System. On additional slides from each case, we performed immunohistochemistry for CD34 as single marker in order to help us form an accurate assessment of CD34_CAFs. Slides that had been stained with immunohistochemistry were mounted with a permanent mounting medium called CV Mount, which was manufactured by Leica Biosystems, New-castle Ltd., in the United Kingdom.

### 2.4. Image Acquisition and Digital Image Analysis (DIA)

**Figure 1:**
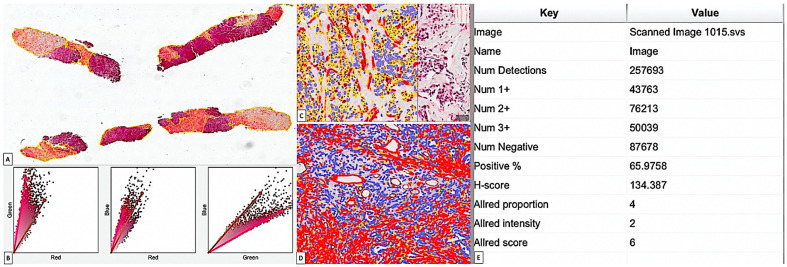
Digital image analysis (DIA) of CAFs using the QuPath open-source platform for bioimage analysis. (**A**) Regions of interest (ROIs) were chosen by using brush tool from QuPath tools. Stroma areas were sharply delineated by the tumor areas. (**B**) Vector stain estimation followed during DIA evaluation, and a visual stain map was generated. (**C**,**D**) Stromal CAFs were counted separately according to the cell parameters and intensity score set by the user. Note that three intensity thresholds (weak-yellow, noted as +1; intermediate-orange, noted as +2; and high-red, noted as +3) were used, and cells with different intensity were reported separately. Stromal and/ or tumor cells detected as negative were highlighted in blue by QuPath_DIA. (**E**) Example of a report table regarding the parameters automatically delivered by QuPath analysis. Allred score from the final report was considered by us as a Stromal Score including both proportion and intensity of total number of cells counted inside selected stromal areas.

IHC samples were scanned using a Grundium OCUS 20 Microscope (Grundium, Tampere, Finland) and stored in the Case Center Slide Library as svs files (3DHistech, Budapest, Hungary). A project has been created by importing all slides stained with CD34/SMA and CD34 alone into QuPath version 0.4.3 [41], an open-source platform for bioimage analysis of microscopic slides, where they were examined using integrated software and its add-ons, such as Fiji and Vascular Analysis, for an accurate evaluation of tumor stromal blood vessels. Usually, QuPath was used in the literature to assess tumor stroma ratio which seems to have a significant prognostic impact [42]. Briefly, we selected 3–5 regions of interests (ROIs) (Figure 1A) from tumor stroma by using the brush tool which gave us the most accurate delineation of tumor stroma from tumor tissue and also excluded stromal blood vessels (assessed by QuPath/Vessels analysis, previously). DIA started with a pre-processing step consisting of estimating stain vectors (Figure 1B). Then, the analysis continued with cell detection by choosing a positive cell detection option and by setting cell parameters and intensity parameters. The detection image was set for an optical density sum with a requested pixel size of 0.5 μm and a cell expansion of 1.988 μm, excluding the nucleus. Intensity threshold parameters comprised the score compartment and three threshold levels evaluated as weak (+1, highlighted with yellow), moderate (+2, highlighted with orange), and high (+3, highlighted in red), with all cells selected in blue being considered negative (Figure 1C,D). During automated scoring, QuPath software provided us the cell count, percentage of positive and negative stromal cells, both density score and intensity score separately, but also a combined Stromal Score (SS, similar to the Allred score, combining intensity and density of positive detected stromal CAFs). Also, an H-score was included in the final evaluation conducted by QuPath analysis (Figure 1E). For our study, we used positive stromal CAF intensity (S_SMA_I and S_CD34_I, respectively), density (S_SMA_D and S_CD34_D, respectively), SS (CD34_SS and SMA_SS), and H-score (CD34_H-Score and SMA_H-Score) for a more accurate evaluation and correlation with clinic-pathologic parameters.

### 2.5. Statistical Analysis

JAMOVI software version 2.3 for macOS devices was used for statistical analysis. DIA-derived data mentioned above were correlated with BC molecular subtypes, menopausal status, immature and mature stromal blood vessels, recurrence, lymphovascular and perineural invasion, and age. A statistical correlation was assessed and considered significant for a *p* value of 0.05 or less.

## 3. Results

### 3.1. General Considerations on Conventional Microscopic Assessment of Stromal CD34 and αSMA in Normal Breast Tissue and BC

**Figure 2:**
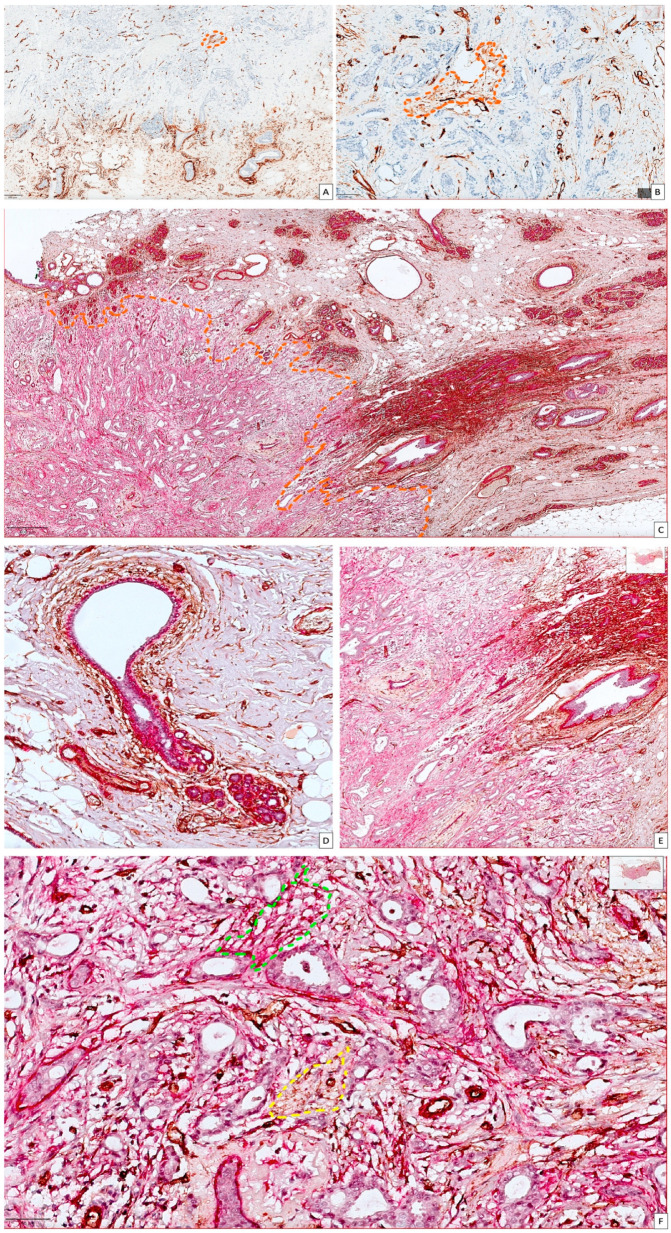
CD34 single (**A**,**B**) and double CD34/αSMA (**C**–**F**) immunohistochemical stains for BC tissues included in our study for highlighting CAFs and normal stromal fibroblast plasticity. (**A**) CD34-positive fibroblast density dramatically decreased from normal (low part of the picture) to malignant tissue (upper part of the picture) but did not completely disappear, being restricted to small intratumor areas (dotted orange line). (**B**) Deep inside BC tissue, small foci of CD34-positive CAFs were detected (dotted orange limited area), but mostly, CD34 positivity was observed inside the tumor blood vessels endothelium. (**C**) CD34/αSMA double-stain (CD34—brown and αSMA—red) sharply delineated differences in between CD34 and αSMA expression in between normal breast tissue (right to the orange dotted line) and BC tissue (left to the dotted line). (**D**) Normal TDLU expressing αSMA restricted to myoepithelial cells which delineate both ductal and acinar components, while CD34 is highly expressed in peri-TDLU stromal fibroblasts but also fibrocytes from dense interlobular stroma. (**E**) Border in between normal and tumor tissue highlighted by the change in the density and intensity of αSMA expression. (**F**) Detail from BC area with a high predominance of αSMA_CAFs (red) inside stroma (multiple areas similar with green dotted lined area) and small and inconstant foci of CD34_CAFs (yellow dotted lined area). Because of these scattered CD34-positive intratumor CAFs, both immunostains were mandatory for a more accurate CAFs heterogeneity evaluation.

A divergent expression of CD34 and αSMA has been observed in the present study. Single IHC for CD34 revealed that positive cell density dramatically decreased from normal to malignant tissue (Figure 2A), remaining positive in tumor vessels’ endothelium and a few CAFs in scattered areas in between tumor cells (Figure 2B). Double IHC for CD34 and αSMA identified high CD34 expression in the stroma surrounding the Terminal Ductal Lobular Unit (TDLU) of the normal breast (Figure 2C). αSMA positivity was found to be restricted to myoepithelial cells from the ductal and lobular structure of a normal mammary gland (Figure 2D) while the inside tumor stroma predominated αSMA-positive CAFs with a high density and staining intensity (Figure 2E). Rare cases presented an intermixed area of αSMA positive CAFs (predominant) and CD34-positive CAFs (scattered) (Figure 2F).

### 3.2. Critical Overview of Digital Image Analysis for BC Stromal CAF Assessment by Using Immunohistochemistry and QuPath Platform

#### 3.2.1. αSMA_CAF Digital Image Analysis

**Figure 3:**
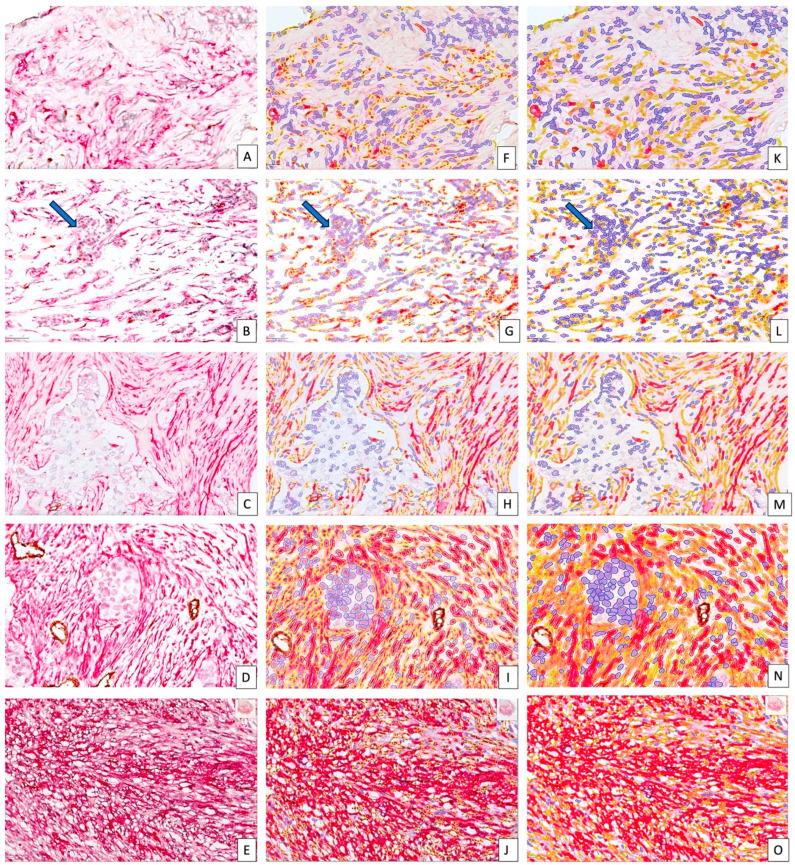
Examples of αSMA Stromal Scores from score 4 (**A**,**F**,**K**), to maximal score 8 (**E**,**J**,**O**). Double-stain CD34/αSMA (DAB, brown/permanent red, red) was highlighted from (**A**–**E**). Stromal αSMA were automatically labeled based on cell and intensity parameter settings in a different manner according to variable intensity (blue—negative, yellow—weak, +1, orange—medium, +2 and red—strong, +3). The method accuracy is high because QuPath analysis may detect tumor-infiltrating cells in the stroma, in between αSMA_CAFs, and label them as negative ((**B**,**G**,**L**), corresponding to a case scored as 5). Groups of infiltrating tumor cells ((**B**), arrow) marked to be negative by automated analysis ((**G**,**L**), arrows). When we compared images (**C**,**D**) by conventional analysis, we were tempted to consider them as having a similar density and intensity of αSMA_CAFs. But this subjective observation was not confirmed by DIA which helped us to classify cases in two different scoring groups based on proportion of different kind of positive cells (**H**,**I**). DIA detected differences between the proportion of weak, moderate, and strong positive cells, and this was extremely important for the final stromal score (**M**–**O**) impossible to differentiate by using conventional assessment). When we compared picture (**F**) with (**G**), we observed differences between the proportions of yellow-, blue-, and red-labeled cells. In (**F**), blue- and yellow-labeled cells predominated while for (**G**), red-labeled cells’ proportion slightly increased together with the yellow-labeled proportion (according to automated measurements given by QuPath analysis). This fact was confirmed better by images (**K**,**L**). The Stromal Score increases once the proportion of red-labeled cells increases ((**H**) with an SS of 6, (**I**) with an SS of 7, and (**J**) with an SS of 8). By using the brush tool from the QuPath menu, we were able to identify CD34-positive vessels from the tumor stroma and exclude them from quantification (**I**,**N**).

We performed both single (for CD34) and double (CD34/αSMA) immunohistochemistry for identification of both CD34_CAFs and αSMA_CAFs. CD34/αSMA double-staining was performed to identify stromal immature and mature blood vessels and for quantification of αSMA_CAFs. It is well known that the manual evaluation of both CD34- or *α*SMA-positive CAFs is practically impossible due usually to a low density (for CD34) and too many high-density (for αSMA) CAFs from the BC stromal compartment. For these cases, the usual interpretation performed manually on IHC slides includes a subjective microscopic appreciation of both CAF density and intensity based on empirical criteria chosen by each pathologist. For example, for cell density three scores are often used: low—often noted as 10–30 positive cells, medium—noted as 30–60 positive cells, and high—noted as 60–100 positive cells on randomly selected BC stroma areas. By applying this algorithm, it seems that cases from Figure 3A,B should be categorized as having low expression for αSMA_CAFs, cases from Figure 3C,D should be categorized as having medium expression, and those from Figure 3E should be categorized as having high expression, undoubtedly. By using the QuPath bioimage analysis platform facilities, we were able to stratify cases into three classes (by giving a density scoring from 3 to 5, based on αSMA_CAF density automatically marked as it has been shown in Figure 3F–J by QuPath software according to cell parameters settings). Also, QuPath has an assessing option of staining intensity by marking cell borders (Figure 3F–J) or cell borders and their cytoplasmic area (Figure 3K–O) with different colored lines specific for each intensity varying from 1 (considering as weak, marked in yellow) to 2 (medium intensity corresponding to an orange line) and 3 (quantified as strong by a red line around positive cells). Cells marked in blue were classified as negative for immunostaining. The final score named by us as Stromal Score (SS) was calculated similar to the Allred score (with a chosen option of not counting nuclei, just cytoplasmic immunoreaction) by the sum of intensity and density for each case and varied from 4 to 8 in the present study (Figure 3A–O). An additional H-Score based on intensity pixel assessment was added as a parameter and varied from 41.366 to 276.149 for αSMA_CAFs. Automated evaluation of αSMA_CAFs revealed different densities and intensities in between BC molecular subtypes.

#### 3.2.2. CD34_CAF Image Analysis

CD34_CAF image analysis performed with QuPath revealed that, despite their extremely low density inside tumor stroma, they may quantify and have a clinical and prognostic impact specific for some BC molecular subtypes. Due to their low number in tumor stroma tissue, it is hard to estimate them on the same slide by a double immunostaining with αSMA because of the high number and intensity staining of αSMA_CAFs which would mask CD34_CAFs.

**Figure 4:**
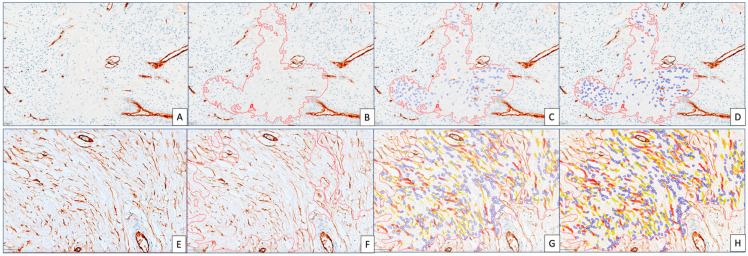
Step by step example of CD34_CAF evaluation in BC tumor stroma by using the same digital analysis method as for αSMA_CAFs. We showed here a case scored as 3 (lowest for CD34_CAFs, (**A**–**D**)) and a case scored as 6 (highest one for CD34_CAFs, (**E**–**H**)).

Thus, we evaluated CD34_CAFs separately on a CD34 simple immunostained specimen. CD34_CAF DIA parameters were lower compared to similar ones for αSMA_CAFs. CD34_CAF density ranged between 2 and 4, with none of the cases reaching a density score of 5. Amongst them, 58.5% were scored as 2 for density; 37.7% had a score of 3; and resting ones had a score of 4. A percentage of 88.7% of cases had an intensity score of 2. CD34_SS ranged between 3 (Figure 4A–D) and 6 (Figure 4E–H), with 94.33% of cases having a CD34_SS of 4 and 5 (56.6% of scores with 4 and 37.73% of scores with 5), with the resting ones being scored as 6. The differential impact of these stromal scores was separately evaluated in the relationship with clinic-pathologic parameters in the next section of this paper.

### 3.3. DIA Impact on Stromal CD34/αSMA_CAF Assessment Related to BC Molecular Subtypes and Clinic-Pathologic Data 

We performed a separate analysis for each BC molecular subtype by correlating parameters obtained from DIA to clinico-pathologic data of patients included in the study as age, menopausal status, survival, TLS, stromal vascular components, invasion, and recurrence. 

#### 3.3.1. CD34/αSMA_CAF Interplay Is Highly Dependent on Age and Strongly Influences Survival and Stromal Components in Luminal A (LA_BC) Subtype

Stromal CD34_CAF density (S_CD34_D) significantly decreased for the LA stromal compartment whereas αSMA_CAF density (S_SMA_D) increased during malignant transformation (*p* = 0.049). It seems that LA subtype is able to acquire a high number of αSMA_CAFs which in turn significantly influenced the decrease of both CD34_SS (*p =* 0.044) and CD34_H-Score (*p* = 0.032), most likely due to a low S_CD34_D. Patients’ age had a significant impact on the αSMA_SS, being inversely but significantly correlated with it (*p* = 0.014) but not with S_SMA_D (*p* = 0.818) or SMA_H-Score (*p* = 0.553). This finding suggested that intensity and less density are influenced by age. Also, αSMA_SS had a direct significant influence on the TLS presence in the tumor stroma from LA_BC subtype (*p* = 0.018). But one of the most interesting findings related to the LA_BC subtype was the direct significant correlation of S_CD34_D (*p* = 0.013) and CD34_H-Score (*p* = 0.022). A low survival rate was correlated with low S_CD34_D for LA_BC. No positive or negative influence on invasion and recurrence has been registered for LA_BC in our study. All data can be summarized by the correlation matrix in Table 1.

#### 3.3.2. αSMA_SS Variability Is Age-Dependent but Also Influences Tumor Grade (G), TLS, and Immature Tumor Blood Vessels Dynamics for Luminal B (LB)_BC Subtype

A direct correlation between age and αSMA_SS has been observed for the LB_BC subtype (*p* = 0.020). Our data showed that 80% of patients >50 years old had a predominant SMA_SS of 7 and 8 compared to 50% for patients <50 years old who had a SMA_SS of 7, but none of them had a Stromal Score of 8. Also, LB_BC is the only BC molecular subtype where G2 is directly influenced by αSMA_SS (*p* = 0.036). Two main important stromal components, TLS and immature tumor stroma blood vessels (IBV_CD34+/SMA-), were influenced by αSMA_SS. All LB_BC cases (100%) with a score of 5 had TLS, but only 50% of cases with a score of 6 kept the TLS presence inside tumor stroma. A percentage of 37.5% of cases scored as 7 retained TLS, but none of the cases scored as 8 presented stromal TLS. This observation was certified by an inverse correlation between αSMA_SS *(p* = 0.009), αSMA_H-Score (*p* = 0.005), and TLS for LB_BC. αSMA_CAFs seem to impair or stop the development of immature stromal tumor blood vessels. We observed that the highest αSMA_SS we have corresponded to the lowest number of IBV_CD34+/SMA- stromal vessels which were counted (an inverse significant correlation with *p* = 0.019). Contrarily, the lowest CD34_H-Score was observed, as a low number of IBV_CD34+/SMA- was counted (direct significant correlation, *p* = 0.009) (Table 2). 

#### 3.3.3. HER2_BC Subtype Is CD34_CAF-Dependent but Not Influenced by αSMA_CAFs

When we assessed HER2_BC, we observed that this subtype is exclusively related to CD34_CAF density and CD34_H-Score. Both parameters had lower values compared to other molecular subtypes, HER2_BC being amongst the BC molecular subtypes with the lowest value of CD34 expression in tumor stroma cells. CD34_CAF density and CD34_H-Score decreased as age increases (*p* < 0.001 for both). About 66.67% of cases had no IBV_CD34+/SMA- stromal vessels. We found that the lack of IBV_CD34+/SMA-stromal vessels significantly was correlated with low CD34_CAF density (*p* < 0.001) and low CD34_H-Score (*p* < 0.001). Interestingly, HER2_BC cases with no immature stromal tumor vessels had no LVI (*p* < 0.001), PnI (*p* < 0.001), or recurrence (*p* < 0.001) (Table 3).

#### 3.3.4. Luminal B-HER2 (LB-HER2) BC Is Influenced by Both Types of CAFs, but Each of Them Had Significant Impact on Different Stromal Vascular Components and Clinic-Pathologic Parameters

**Figure 5:**
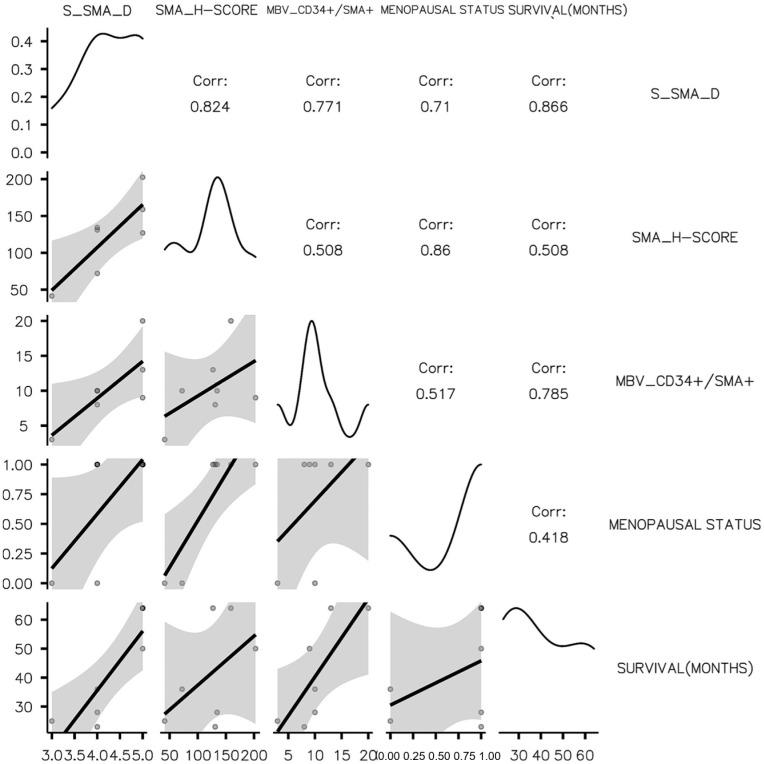
Plot representation of correlation matrix for the LB_HER2 subtype related to αSMA_CAFs.

**Figure 6:**
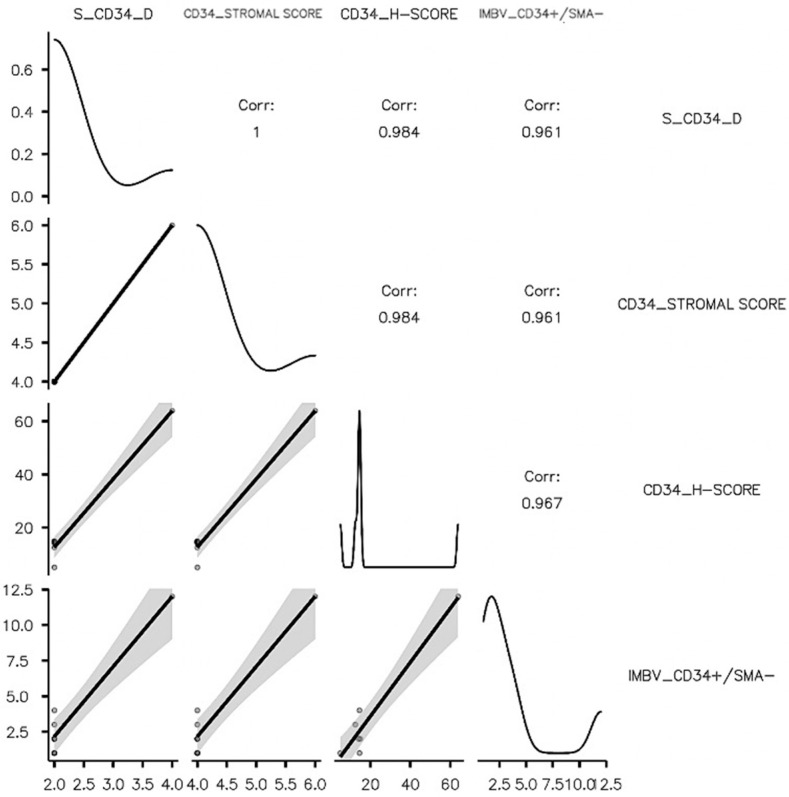
Plot representation of correlation matrix for the LB_HER2 subtype related to CD34_CAFs.

Mature (MBV_CD34+/αSMA+) and immature (IBV_CD34+/αSMA-) stromal vessel density was assessed previously, and data were registered and used to study the influence of tumor stroma CAFs on their variability. As for HER2_BC, LB-HER2 stromal IBV_CD34+/αSMA- density was highly influenced by CD34_CAFs, but, for this subtype, all three DIA parameters (S_CD34_D, CD34_SS, and CD34-H-Score) were strongly and directly correlated with IBV_CD34+/αSMA- density (*p* < 0.001). Low CD34_CAF density supported a low density of immature stromal vessels. These findings suggest that CD34_CAFs may be tumor stromal cellular components with the potential for mesenchymal to endothelial transition followed by an angiogenic switch. Contrarily, we reported here that S_αSMA_D was directly correlated with MBV_CD34+/αSMA+ stromal vessel density (*p* = 0.043). A high survival rate was significantly correlated with high S_αSMA_D in tumor stroma (*p* = 0.012). Menopausal women had a significantly higher αSMA_H-Score compared to pre-menopausal patients (*p* = 0.013) for the LB_HER2 BC subtype. A correlation matrix and correlation plot sustaining the above presented data may be seen in the overview in Table 1 and Figure 5. All detailed data related to CD34_CAF impact on LB_HER2 BC subtype may be seen in Table 4 and Table 5 and Figure 5 and Figure 6.

We consider these data to have high originality due to the lack of reports related to the LB_HER2 BC subtype about stromal cell heterogeneity and their impact on survival and prognosis.

#### 3.3.5. CD34_CAFs Are Key Players of TNBC_BC Stroma Influencing G, NPI, Invasion, Recurrence, and Survival

Values of the Nottingham Prognostic Index (NPI) and G seem to be CD34_CAF-dependent on TNBC_BC. An NPI value of 7 corresponded to a CD34_SS of 4 while an NPI value of 8 significantly overlapped with a CD34_SS of 5 and thus gave us a global significant correlation between NPI and CD34_SS for a value of *p* = 0.009. All G2 cases had a CD34_SS of 4 while 71.42% of G3 cases had a CD34_SS of 5. To these correspondences, a significant correlation has been reported (*p* = 0.024). For survival and menopausal status, a partial, low-significance correlation has been registered related to CD34_SS and CD34_H-Score. PnI and recurrence were highly dependent on the presence of CD34_CAFs for a correlation with all three parameters (*p* = 0.009 for S_CD34_D; *p* = 0.032 for CD34_SS; and *p* = 0.002 for CD34_H-Score), while LVI had a significant correlation with CD34_SS only. We did not detect any significant impact of CD34_CAFs on the tumor stromal vascular compartment for the TNBC BC subtype based on DIA parameters.

## 4. Discussion

The impact of the tumor–stroma ratio on prognosis and therapy response is a relatively new topic in BC research field [32,33,43] in recent years. Its diagnostic and prognostic value increased by association of tumor stroma ratio with other clinic-pathologic parameters such as multiparametric MRI findings [43], oncogenic proteins [44], axillary lymph node metastasis [45], or TNM staging [46]. The tumor–stroma ratio was evaluated using different DIA software, but it seems that one of the favorites is the QuPath analysis platform due to its facilities covering a wide spectrum of needs for researchers who assess microscopic images with an emphasis on tumor stroma [38,39,47]. We also chose the QuPath platform due to its tools’ ability to sharply detect and label stromal fibroblasts and automatically perform stromal scoring and H-Score. By its versatile functions for choosing cell parameters but also three thresholds for staining intensity, the QuPath platform provided us with a unique tool for detection and quantification of stromal CAFs based on both morphological and immunohistochemical parameters. These proved to be very useful for our following analysis of CAF impact on clinic-pathologic parameters due to several correlations found in the present study.

Divergent expression of CD34 and αSMA in normal breast stromal fibroblasts and tumor stroma CAFs was previously observed and reported by several authors [6,19,48,49]. CD34 positive fibroblasts’ presence was highly associated with normal breast stroma, while αSMA-positive CAFs were intensely discussed as being specific to the BC tumor stroma [6,19,48,49]. For BC where CD34-positive stromal cells are present inside the tumor stroma, some authors suggested that CD34-positive BC stromal cells/telocytes are precursors for αSMA-positive stromal CAFs [27], and this may explain divergent expression of two markers from the ability of CD34-positive stromal cells to dedifferentiate into αSMA-positive CAFs. Also, CD34-positive stromal cells have been reported to be a source of mesenchymal cells [25], which may have the ability to differentiate into several cell types from endothelial cells [24] to myofibroblasts in BC stroma [50].

Because of CD34-positive CAFs’ low density in the BC stromal compartment, their conventional microscopic objective assessment is practically impossible. Several BC studies reported the lack of CD34-positive CAFs by using conventional microscopy instead of using automated quantification. On the other hand, the CD34 antigen is also expressed in the endothelium of tumor stromal blood vessels and is highly associated with tumor angiogenesis instead of its CAF expression [51,52,53]. The above evidence supports the prognostic role of CD34-positive CAFs as versatile cells with a high ability for dedifferentiation in various heterogeneous cells from stromal to malignant ones. This uncontrolled and less elucidated dedifferentiation may be a trigger for therapy resistance development or for the development of malignant cells with a high ability for invasion and metastasis. An accurate and correct assessment of CD34_CAFs is not possible without digital image analysis. Thus, we consider their assessment by using IHC and DIA to be necessary as a routine procedure during initial microscopic evaluation of any BC case to predict a potential bad prognosis or development of resistance to therapy. Our recommendation is supported by our findings and by some of the literature data pointing out CD34_CAFs’ role in BC. Recently, Westhoff and collaborators reported the prognostic relevance of CD34_CAF loss in BC [17]. Their semiquantitative conventional grading results referred to invasive lobular carcinoma (ILC) as being related to its morphological subtypes, the presence of metastasis, and pTNM staging parameters without any mention of BC molecular subtypes. The present study partially confirmed their results but is closely related to BC molecular subtypes. We found that a low CD34 _CAF density was significantly correlated with a low survival rate for the LA subtype. Other authors previously mentioned that CD34_CAFs’ presence may exert a positive role in various tumor types such as lung cancer [54], pancreatic neoplastic lesions [55], or cervical cancer [56], most likely due to their involvement in tumor stroma remodeling with no relation to prognostic or clinical parameters. Thus, a CD34_CAF variability assessment may stratify BC patients with the LA subtype into different survival rate subgroups with a possible different response to therapy and disease-free survival.

There are several data points about αSMA_CAFs’ involvement in the development of resistance to Trastuzumab [57,58,59], but there are none about CD34_CAFs’ influence on the HER2 subtype of BC. By applying DIA analysis of IHC-stained slides, we found that the lowest CD34_CAFs_D was registered inside the stroma of the HER2-positive subtype. This seems to influence the development of a low number of tumor stroma blood vessels of an immature type which in turn may limit LVI and PnI according to our findings. No data about a significant correlation between CD34_CAFs_D, tumor stroma vessels, and invasion is available at this time in the literature for BC, and thus, our findings will need further studies for a complete characterization of CD34_CAFs’ impact on therapy and prognosis for the HER2 subtype. Indirect evidence which may support our correlation between low CD34_CAFs_D and low immature tumor stroma blood vessel (IBV_CD34+/αSMA-) density was published by San Martin et al. in 2014. They hypothesized that CD 34_CAFs’ recruitment by the tumor stroma is achieved from the stromal microvasculature [60]. As low as microvascular density is, low CD34_CAFs_D is observed as we also reported here. Interestingly, this interrelation between IBV_CD34+/αSMA- and CD34_CAFs was also found for the LB and LB-HER2 subtypes but not for TNBC. The strongest significant correlation between all CD34_CAFs parameters and IBV_CD34+/αSMA- was observed for the LB-HER2 subtype in the present study. All DIA-assessed CD34_CAFs parameters showed a strong correlation with stromal immature blood vessels. Based on our analysis, we may partially confirm the reactive vascular hypothesis of San Martin et al. [60], with the novelty of the present study being that this confirmation characterized HER2 and LB-HER2 BC subtypes only.

But the highest significance of CD34_CAF DIA assessment was noticed for TNBC_BC in the present study not only with impact on other stromal components but also related to tumor grade and NPI. Recently, Wu et al. [61] described the TNBC stroma heterogeneity related to the presence of four types of CAFs and reported their influence on tumor immune evasion ability but not on invasion or other prognostic markers. They defined CD34_CAFs as inflammatory CAFs (iCAFs) and proved that they are distributed far from malignant areas intermixed with a heterogeneous population of inflammatory cells [56]. In the same paper, the authors mentioned the iCAFs related to the enrichment of growth factor gene ontologies with a strong upregulation of crosstalk via the FGF, BMP, HGF, and IGF1 pathways through the corresponding receptors distributed in malignant cells and endothelial cells. This crosstalk is known to induce BC cell proliferation and invasion [56] in TNBC_BC. Their findings support our significant correlations found between CD34_CAF DIA parameters (density, stromal score, and H-Score) and G, NPI, LVI, and PnI specific to TNBC_BC. To the best of our knowledge, we were not able to find any literature data which may support CD34_CAFs’ association with microscopic and/or clinico-pathologic parameters in the TNBC_BC subgroup, specifically most likely due to the lack of CD34_CAFs in a digital image analysis program. This is one more reason sustaining DIA CD34_CAFs’ utility for the evaluation improvement of TNBC_BC patients. The same study by Wu et al. stated that the classical angiogenic pathways were enriched for iCAFs, and thus, iCAFs induce tumor neovascularization. This also may support our findings related to the interrelation between CD34_CAFs (iCAFs) and IBV_CD34+/SMA- for some BC molecular subtypes [61].

Myofibroblast-like CAFs (myCAFs) are another type of CAF described in the BC stromal compartment, but, compared with iCAFs, myCAFs are located at the tumor–stroma interface and invasion front, close to the tumor area [56]. For IHC, the most common marker used to identify myCAFs is the αSMA antibody which we also used in the present study. myCAF marker αSMA accumulation in the tumor stromal fibroblast has a poor prognostic significance for BC patients [62,63]. αSMA_CAFs are responsible not only for poor prognosis in primary malignancy, but also, they are also recently described as being responsible for poor prognosis and a high recurrence rate of brain metastases [64]. These effects were associated especially with αSMA and PDGFR-β expression in stromal myofibroblast-like cells. αSMA_CAFs induce tumor stroma remodeling by targeting several stromal components from stromal vessels to collagen synthesis, inflammatory cells, and tertiary lymphoid structures (TLSs). αSMA_CAFs modulate the immune tumor microenvironment of several cancer types [65], but the interplay in between αSMA_CAFs and TLSs is less elucidated in malignancies, including breast cancer also. 

The prevalence of stromal myofibroblasts (i.e., αSMA-positive fibroblasts) in human breast cancers is related to aggressive adenocarcinomas and predicts human disease recurrence [66,67]. Costa et al. identified four types of CAFs in human breast cancer stroma (CAF-S1 to CAF-S4) [68], but only two of them are described to be highly positive for αSMA (CAF-S1 and CAF-S4). CAF-S3 was characterized to having negative to low expression of αSMA_CAFs [68]. Thus, differences in between αSMA immune expression must be quantified by using the digital image analysis. This may be a simple method applied to differentiate between various αSMA_CAF types in routine IHC combined with DIA at the first evaluation of BC specimens. Differential assessment of αSMA expression intensity for CAFs (performed by DIA to obtain stromal score like has been described in the present study) may suggest CAFs’ heterogeneity from BC stroma and may recommend additional tests to predict poor prognosis and/or recurrence by pathologists.

It has been proved that different CAF types are heterogeneously distributed in between BC molecular subtypes [63]. This is in line with our findings. We reported here not only differences between CD34_CAFs and αSMA_CAFs’ impact in BC but also the interrelation with other stromal components as tumor stroma vasculature and immune cells or clinic-pathologic parameters. One new finding arising from the present study was the significant association of age with αSMA_SS and/or αSMA_H-Score for LA_BC and LB_BC subtypes. These findings suggest that age-related differences may influence the BC patient’s stroma reaction to malignant conditions based on CD34_CAFs and αSMA_CAFs.

The impact of CAFs on vasculature and the tumor stroma immune microenvironment is less studied. By using double immunostaining for endothelial (CD34) and perivascular (αSMA) markers, we were able to assess not only stromal vessels’ maturation grade and types but also their interrelation with αSMA_CAFs. In the same paper by Costa et al., the authors pointed out the differences between CAF-S1 and CAF-S4 at the transcriptomic level, suggesting a different role of these two types of αSMA-expressing CAFs [68]. This may explain in part our contradictory results found for αSMA_CAFs’ interrelations with TLSs between LA_BC and LB_BC subtypes. For LA_BC, our results suggest that αSMA_CAFs stimulate TLS formation by a significant direct correlation between these two parameters. These CAFs present in LA-BC are most probably the CAF-S1 type known for its ability to attract and instruct immune cells through a phenotype such as that of cells composing TLSs, and by this mechanism they stimulate TLS organization. Until the present study, this was proved for TNBC_BC but not for LA_BC. Based on our findings, further studies will be needed to elucidate if TLSs’ presence in LA_BC may split this molecular subtype into two subgroups with a possible different response to antitumor immune therapy. The interrelation between CAFs and TLSs has been also reported for lung cancer [69]. Bonneau et al. [70] created a CAF types of distribution map for luminal A and B early breast cancer, linking their accumulation to recurrence. They reported that CAF-S1 enrichment of early luminal BC is a risk factor for recurrence, being independent of age and epithelial, immune, and vascular features. Our data are partially in contradiction with previous findings. We found that αSMA_CAFs in LA_BC are highly and significantly related to the TLS presence (an immune component of tumor stroma) and influenced by age, but we did not find any impact on recurrence and invasion for LA_BC. The interrelation between age and αSMA_CAF Stromal Score was kept for LB_BC also. But the most original findings of the present paper may be considered that αSMA_CAFs seem to negatively influence tumor stroma immature vessels and TLS development in LB_BC. We found that TLSs’ presence drastically decreased with increased αSMA_SS, suggesting an inverse correlation between them for LB_BC compared to LA_BC where we found a direct significant correlation. The discrepancies between the two types of luminal BC may be explained in part by the presence of different CAF phenotypes. CAF-S1 and CAF-S4 are two types of fibroblasts which are both positive for αSMA, but several differences related to traits other than their markers have been reported [68]. CAF-S1 has upregulated genes involved in cell adhesion, extracellular matrix organization, and immune response while CAF-S4 is responsible for muscle contraction, regulation of the actin cytoskeleton, and oxidative metabolism [68].

Based on the above evidence related to distinct CAF functions, we may speculate that CAF-S1’s associated function as a promoter of TLS immune cells’ development may sustain our findings related to a direct correlation between TLSs and αSMA-CAFs found for LA_BC. Contrarily, an inverse correlation found between the same factors for LB_BC may suggest the presence of another CAF subset positive for αSMA, CAF-S4. This subset has no immunogenic role, but it is called a perivascular type according to its phenotype and function [68,71]. The non-immunogenic role of CAF-S4 sustains the inverse correlation found in the present study between TLSs and αSMA-CAFs. Their perivascular phenotype may suggest their contribution to a rapid maturation of tumor stroma blood vessels during tumor angiogenesis. This is in accordance with our findings for LB_BC where the inverse correlation between IBV_CD34+/SMA- stromal vessels and αSMA-SS has been reported. Another hypothesis related to these two inverse correlations found for LB_BC may be the mechanical impact of CAF-S4 on tumor stroma components related to their certified contractility ability. Changes in the tumor stroma stiffness related to CAF-S4 contractility may impair stromal immune cell trafficking and vascular network expansion.

The interrelation between TLSs and tumor stroma blood vessels was recently reported by our team [72] and together with the findings from the present study may sustain the expansion of BC stroma research by integrating as many stromal components as we have for a better understanding of tumor stroma heterogeneity.

A limited number of papers assessed both αSMA_CAF and CD34_CAF interplay in breast cancer stroma [73], with most of them being related to the diagnosis of breast pathologic conditions [74,75,76] other than invasive breast cancer molecular subtypes. This may be the main reason why CD34_CAFs’ role in highly aggressive BC subtypes such as HER2 and TNBC is underestimated in its relationship with other clinic-pathologic parameters and stromal vascular and immune components.

The identification of novel CAF biomarkers is a continuously growing research field for different cancers [77,78,79]. Similar to CD34_CAFs which have the ability to transdifferentiate into endothelial cells which may favor new blood vessel development, podoplanin-positive CAFs [80], which recently received more attention in breast cancer, may be assessed as potential precursors for the development of new tumor lymphatics, for promoting tumor progression invasion and metastasis, or for serving as a target for personalized therapy [81].

Due to the small geographic area where BC cases were collected and due to the lack of a national database for research, the number of cases selected for the present study might seem small. This could be considered as a limitation of the present study, but it may be partially counterbalanced by the new original findings with special emphasis on CD34_CAFs’ different impact on BC molecular subtypes and both CD34_CAFs’ and SMA_CAFs’ interrelation with other stromal components. The validation of our results by further studies on a large cohort of cases could be developed starting from our data. 

## 5. Conclusions

The present paper demonstrated that both αSMA_CAFs and CD34_CAFs have a significant impact on tumor invasion, metastasis, recurrence, and survival differently in between BC molecular subtypes through their interplay with other stromal components (as immune cells and vascular network), being influenced by age, menopausal status, tumor grade, and NPI. Our results together with previously published data strongly suggest a stringent need for BC specimens’ initial evaluation improvement by using IHC techniques combined with DIA methods applied not only to tumor epithelial components assessment but also to cellular heterogeneity of BC stromal fibroblasts. In this light, CD34_CAF assessment should receive similar attention as αSMA_CAFs have already received. Our data revealed that, despite their low density in tumor stroma, CD34_CAFs have a significant influence on LVI, PnI, NPI, G, recurrence, and survival in some of the most aggressive BC subtypes such as HER2 and TNBC. Scattered and elusive data found in the literature related to CD34_CAFs’ impact may be due to the lack of using DIA for their assessment. We proved here that CD34-CAFs should be mandatory for assessment together with αSMA_CAFs by using special DIA software because they may influence important clinic-pathologic parameters dependent on BC molecular subtypes. The data about the potential impact of CD34_CAFs on BC clinic-pathologic parameters highlighted on the present paper, together with our findings about CD34_CAFs’ and αSMA_CAFs’ impact on the LB-HER2 BC molecular subtype may be considered the most original parts of the present study.

## Figures and Tables

**Table 1:** Correlation matrix for LA BC showing significant correlations for this BC molecular subtype.

		S_SMA_I	S_SMA_D	SMA_STROMAL SCORE	SMA_H-SCORE	S_CD34_I	S_CD34_D
SMA_STROMAL SCORE	Pearson’s r	0.693 *	0.189	—			
	*p*-value	0.038	0.626	—			
	95% CI Upper	0.929	0.758	—			
	95% CI Lower	0.054	−0.543	—			
	Spearman’s rho	0.709 *	0.189	—			
	*p*-value	0.032	0.626	—			
	Kendall’s Tau B	0.685 *	0.189	—			
	*p*-value	0.045	0.593	—			
SMA_H-SCORE	Pearson’s r	−0.079	0.000	−0.095	—		
	*p*-value	0.839	1.000	0.808	—		
	95% CI Upper	0.617	0.664	0.607	—		
	95% CI Lower	−0.706	−0.664	−0.714	—		
	Spearman’s rho	0.040	0.000	0	—		
	*p*-value	0.919	1.000	1	—		
	Kendall’s Tau B	0.075	0.000	0	—		
	*p*-value	0.801	1.000	1.000	—		
S_CD34_I	Pearson’s r	−0.105	−0.286	−0.378	−0.113	—	
	*p*-value	0.788	0.456	0.316	0.772	—	
	95% CI Upper	0.601	0.467	0.382	0.596	—	
	95% CI Lower	−0.719	−0.798	−0.833	−0.723	—	
	Spearman’s rho	−0.124	−0.286	−0.378	0.000	—	
	*p*-value	0.751	0.456	0.316	1.000	—	
	Kendall’s Tau B	−0.120	−0.286	−0.378	0.000	—	
	*p*-value	0.726	0.419	0.285	1.000	—	
S_CD34_D	Pearson’s r	0.490	−0.668 *	0.000	0.363	0.134	—
	*p*-value	0.180	0.049	1.000	0.337	0.732	—
	95% CI Upper	0.871	−0.007	0.664	0.828	0.733	—
	95% CI Lower	−0.258	−0.923	−0.664	−0.397	−0.582	—
	Spearman’s rho	0.551	−0.624	0.100	0.329	0.113	—
	*p*-value	0.124	0.073	0.798	0.388	0.771	—
	Kendall’s Tau B	0.502	−0.600	0.096	0.272	0.109	—
	*p*-value	0.127	0.078	0.777	0.355	0.748	—
CD34_STROMAL SCORE	Pearson’s r	0.341	−0.679 *	−0.189	0.234	0.607	0.869 **
	*p*-value	0.370	0.044	0.626	0.544	0.083	0.002
	95% CI Upper	0.819	−0.026	0.543	0.778	0.906	0.972
	95% CI Lower	−0.418	−0.926	−0.758	−0.509	−0.095	0.483
	Spearman’s rho	0.403	−0.661	−0.097	0.301	0.606	0.855 **
	*p*-value	0.283	0.053	0.804	0.430	0.084	0.003
	Kendall’s Tau B	0.344	−0.617	−0.091	0.225	0.566	0.825 **
	*p*-value	0.281	0.062	0.784	0.433	0.087	0.01
CD34_H-SCORE	Pearson’s r	0.328	−0.711 *	−0.233	0.449	0.252	0.948 ***
	*p*-value	0.389	0.032	0.546	0.225	0.514	<0.001
	95% CI Upper	0.815	−0.089	0.510	0.858	0.785	0.989
	95% CI Lower	−0.430	−0.934	−0.777	−0.306	−0.495	0.768
	Spearman’s rho	0.347	−0.574	−0.092	0.294	0.313	0.921 ***
	*p*-value	0.361	0.106	0.814	0.442	0.412	<0.001
	Kendall’s Tau B	0.268	−0.504	−0.081	0.171	0.275	0.840 **
	*p*-value	0.372	0.104	0.795	0.527	0.376	0.005
TLS	Pearson’s r	0.367	0.357	0.756 *	−0.132	−0.286	−0.267
	*p*-value	0.331	0.345	0.018	0.735	0.456	0.487
	95% CI Upper	0.829	0.825	0.945	0.583	0.467	0.483
	95% CI Lower	−0.393	−0.402	0.184	−0.732	−0.798	−0.791
	Spearman’s rho	0.371	0.357	0.756 *	−0.104	−0.286	−0.170
	*p*-value	0.325	0.345	0.018	0.791	0.456	0.662
	Kendall’s Tau B	0.359	0.357	0.756 *	−0.089	−0.286	−0.164
	*p*-value	0.294	0.312	0.033	0.770	0.419	0.630

Note: * *p* < 0.05—statistically significant; ** *p* < 0.01—highly statistically significant; *** *p* < 0.0001—strongest statistical significance.

**Table 2:** Correlation matrix for LB BC subtype showing significant correlations for this BC molecular subtype related to immune and vascular stromal components and SMA_CAFs digital parameters.

		H-SCORE	S_SMA_I	S_SMA_D	S-SCORE	IMBV_CD34+/SMA-
S_SMA_I	Pearson’s r	0.506 *	—			
	*p*-value	0.027	—			
	95% CI Upper	0.781	—			
	95% CI Lower	0.068	—			
	Spearman’s rho	0.388	—			
	*p*-value	0.101	—			
	Kendall’s Tau B	0.338	—			
	*p*-value	0.073	—			
S_SMA_D	Pearson’s r	0.765 ***	0.056	—		
	*p*-value	<0.001	0.821	—		
	95% CI Upper	0.905	0.497	—		
	95% CI Lower	0.476	−0.409	—		
	Spearman’s rho	0.871 ***	0.092	—		
	*p*-value	<0.001	0.707	—		
	Kendall’s Tau B	0.745 ***	0.099	—		
	*p*-value	<0.001	0.654	—		
S-SCORE	Pearson’s r	0.870 ***	0.685 **	0.731 ***	—	
	*p*-value	<0.001	0.001	<0.001	—	
	95% CI Upper	0.949	0.869	0.890	—	
	95% CI Lower	0.687	0.335	0.414	—	
	Spearman’s rho	0.895 ***	0.603 **	0.813 ***	—	
	*p*-value	<0.001	0.006	<0.001	—	
	Kendall’s Tau B	0.779 ***	0.555 **	0.722 ***	—	
	*p*-value	<0.001	0.008	<0.001	—	
IMBV_CD34+/SMA-	Pearson’s r	−0.378	−0.434	−0.370	−0.532 *	—
	*p*-value	0.111	0.064	0.119	0.019	—
	95% CI Upper	0.092	0.026	0.101	−0.103	—
	95% CI Lower	−0.71	−0.742	−0.706	−0.794	—
	Spearman’s rho	−0.354	−0.346	−0.352	−0.446	—
	*p*-value	0.137	0.147	0.139	0.056	—
	Kendall’s Tau B	−0.187	−0.267	−0.293	−0.339	—
	*p*-value	0.285	0.179	0.149	0.079	—
TLS	Pearson’s r	−0.617 **	−0.519 *	−0.396	−0.579 **	0.540 *
	*p*-value	0.005	0.023	0.094	0.009	0.017
	95% CI Upper	−0.227	−0.085	0.071	−0.170	0.798
	95% CI Lower	−0.837	−0.788	−0.720	−0.818	0.113
	Spearman’s rho	−0.603 **	−0.505 *	−0.380	−0.561 *	0.544 *
	*p*-value	0.006	0.028	0.108	0.013	0.016
	Kendall’s Tau B	−0.505 *	−0.482 *	−0.372	−0.519 *	0.479 *
	*p*-value	0.010	0.032	0.107	0.017	0.021
G	Pearson’s r	0.280	0.367	0.331	0.484 *	−0.271
	*p*-value	0.245	0.122	0.167	0.036	0.261
	95% CI Upper	0.652	0.704	0.682	0.769	0.208
	95% CI Lower	−0.199	−0.104	−0.145	0.038	−0.646
	Spearman’s rho	0.289	0.345	0.364	0.466 *	−0.421
	*p*-value	0.230	0.148	0.126	0.044	0.073
	Kendall’s Tau B	0.238	0.327	0.348	0.434 *	−0.370
	*p*-value	0.221	0.140	0.125	0.043	0.070
AGE	Pearson’s r	0.354	0.358	0.358	0.527 *	−0.459 *
	*p*-value	0.137	0.133	0.132	0.020	0.048
	95% CI Upper	0.696	0.699	0.699	0.792	−0.006
	95% CI Lower	−0.119	−0.115	−0.115	0.096	−0.756
	Spearman’s rho	0.339	0.308	0.345	0.474 *	−0.287
	*p*-value	0.156	0.199	0.148	0.040	0.234
	Kendall’s Tau B	0.226	0.238	0.287	0.360	−0.235
	*p*-value	0.190	0.222	0.151	0.057	0.194

Note. * *p* < 0.05—statistically significant, ** *p* < 0.01—highly statistically significant, *** *p* < 0.001—strongest statistical significance.

**Table 3:** Raw data about SMA_CAF impact on HER2 BC subtype.

		S_SMA_I	SMA_STROMAL SCORE	SMA_H-SCORE	CD34_H-SCORE	S_CD34_D	AGE	IMBV_CD34+/SMA-
SMA_STROMAL SCORE	Pearson’s r	1.000 ***	—					
	*p*-value	<0.001	—					
	Spearman’s rho	1.000 ***	—					
	*p*-value	<0.001	—					
	Kendall’s Tau B	1.000	—					
	*p*-value	0.157	—					
SMA_H-SCORE	Pearson’s r	0.814	0.814	—				
	*p*-value	0.395	0.395	—				
	Spearman’s rho	0.866	0.866	—				
	*p*-value	0.333	0.333	—				
	Kendall’s Tau B	0.816	0.816	—				
	*p*-value	0.221	0.221	—				
CD34_H-SCORE	Pearson’s r	−0.500	−0.500	−0.910	—			
	*p*-value	0.667	0.667	0.272	—			
	Spearman’s rho	−0.500	−0.500	−0.866	—			
	*p*-value	0.667	0.667	0.333	—			
	Kendall’s Tau B	−0.500	−0.500	−0.816	—			
	*p*-value	0.480	0.480	0.221	—			
S_CD34_D	Pearson’s r	−0.500	−0.500	−0.910	1.000 ***	—		
	*p*-value	0.667	0.667	0.272	<0.001	—		
	Spearman’s rho	−0.500	−0.500	−0.866	1.000 ***	—		
	*p*-value	0.667	0.667	0.333	<0.001	—		
	Kendall’s Tau B	−0.500	−0.500	−0.816	1.000	—		
	*p*-value	0.480	0.480	0.221	0.157	—		
AGE	Pearson’s r	0.500	0.500	0.910	−1.000 ***	−1.000 ***	—	
	*p*-value	0.667	0.667	0.272	<0.001	<0.001	—	
	Spearman’s rho	0.500	0.500	0.866	−1.000 ***	−1.000 ***	—	
	*p*-value	0.667	0.667	0.333	<0.001	<0.001	—	
	Kendall’s Tau B	0.500	0.500	0.816	−1.000	−1.000	—	
	*p*-value	0.480	0.480	0.221	0.157	0.157	—	
MBV_CD34+/SMA+	Pearson’s r	0.000	0.000	−0.581	0.866	0.866	−0.866	
	*p*-value	1.000	1.000	0.605	0.333	0.333	0.333	
	Spearman’s rho	0.000	0.000	−0.500	0.866	0.866	−0.866	
	*p*-value	1.000	1.000	1.000	0.333	0.333	0.333	
	Kendall’s Tau B	0.000	0.000	−0.333	0.816	0.816	−0.816	
	*p*-value	1.000	1.000	1.000	0.221	0.221	0.221	
IMBV_CD34+/SMA-	Pearson’s r	−0.500	−0.500	−0.910	1.000 ***	1.000 ***	−1.000 ***	—
	*p*-value	0.667	0.667	0.272	<0.001	<0.001	<0.001	—
	Spearman’s rho	−0.500	−0.500	−0.866	1.000 ***	1.000 ***	−1.000 ***	—
	*p*-value	0.667	0.667	0.333	<0.001	<0.001	<0.001	—
	Kendall’s Tau B	−0.500	−0.500	−0.816	1.000	1.000	−1.000	—
	*p*-value	0.480	0.480	0.221	0.157	0.157	0.157	—
LVI	Pearson’s r	−0.500	−0.500	−0.910	1.000 ***	1.000 ***	−1.000 ***	1.000 ***
	*p*-value	0.667	0.667	0.272	<0.001	<0.001	<0.001	<0.001
	Spearman’s rho	−0.500	−0.500	−0.866	1.000 ***	1.000 ***	−1.000 ***	1.000 ***
	*p*-value	0.667	0.667	0.333	<0.001	<0.001	<0.001	<0.001
	Kendall’s Tau B	−0.500	−0.500	−0.816	1.000	1.000	−1.000	1.000
	*p*-value	0.480	0.480	0.221	0.157	0.157	0.157	0.157
PnI	Pearson’s r	−0.500	−0.500	−0.910	1.000 ***	1.000 ***	−1.000 ***	1.000 ***
	*p*-value	0.667	0.667	0.272	<0.001	<0.001	<0.001	<0.001
	Spearman’s rho	−0.500	−0.500	−0.866	1.000 ***	1.000 ***	−1.000 ***	1.000 ***
	*p*-value	0.667	0.667	0.333	<0.001	<0.001	<0.001	<0.001
	Kendall’s Tau B	−0.500	−0.500	−0.816	1.000	1.000	−1.000	1.000
	*p*-value	0.480	0.480	0.221	0.157	0.157	0.157	0.157
R	Pearson’s r	−0.500	−0.500	−0.910	1.000 ***	1.000 ***	−1.000 ***	1.000 ***
	*p*-value	0.667	0.667	0.272	<0.001	<0.001	<0.001	<0.001
	Spearman’s rho	−0.500	−0.500	−0.866	1.000 ***	1.000 ***	−1.000 ***	1.000 ***
	*p*-value	0.667	0.667	0.333	<0.001	<0.001	<0.001	<0.001
	Kendall’s Tau B	−0.500	−0.500	−0.816	1.000	1.000	−1.000	1.000
	*p*-value	0.480	0.480	0.221	0.157	0.157	0.157	0.157

*** *p* < 0.001—strongest statistical significance.

**Table 4:** Correlation matrix for LB_HER2 BC showing a significant correlation between αSMA_CAFs parameters derived from DIA with tumor stroma mature blood vessels (MBV_CD34+/SMA+), survival, and menopausal status.

		S_SMA_D	SMA_H-SCORE	MBV_CD34+/SMA+
SMA_H-SCORE	Pearson’s r	0.824 *	—	
	*p*-value	0.023	—	
	95% CI Upper	0.973	—	
	95% CI Lower	0.186	—	
	Spearman’s rho	0.694	—	
	*p*-value	0.083	—	
	Kendall’s Tau B	0.620	—	
	*p*-value	0.071	—	
MBV_CD34+/SMA+	Pearson’s r	0.771 *	0.508	—
	*p*-value	0.043	0.244	—
	95% CI Upper	0.964	0.912	—
	95% CI Lower	0.042	−0.397	—
	Spearman’s rho	0.701	0.342	—
	*p*-value	0.080	0.452	—
	Kendall’s Tau B	0.635	0.293	—
	*p*-value	0.068	0.362	—
MENOPAUSAL STATUS	Pearson’s r	0.710	0.860 *	0.517
	*p*-value	0.074	0.013	0.235
	95% CI Upper	0.953	0.979	0.914
	95% CI Lower	−0.092	0.303	−0.387
	Spearman’s rho	0.683	0.791 *	0.399
	*p*-value	0.091	0.034	0.375
	Kendall’s Tau B	0.653	0.690	0.354
	*p*-value	0.094	0.053	0.329
SURVIVAL (MONTHS)	Pearson’s r	0.866 *	0.508	0.785 *
	*p*-value	0.012	0.245	0.036
	95% CI Upper	0.980	0.912	0.967
	95% CI Lower	0.326	−0.397	0.079
	Spearman’s rho	0.856 *	0.378	0.836 *
	*p*-value	0.014	0.403	0.019
	Kendall’s Tau B	0.751 *	0.293	0.650 *
	*p*-value	0.031	0.362	0.046

S_SMA_D—DIA-derived density of αSMA_CAFs; SMA_H-Score—histological score automated calculated by QuPath platform. * *p* < 0.05—statistically significant.

**Table 5:** Correlation matrix for LB_HER2 BC showing a significant correlation between CD34_CAFs parameters derived from DIA with tumor stroma immature blood vessels (IMBV_CD34+/SMA+), survival, and menopausal status.

		S_CD34_D	CD34_STROMAL SCORE	CD34_H-SCORE
CD34_STROMAL SCORE	Pearson’s r	1.000 ***	—	
	*p*-value	<0.001	—	
	95% CI Upper	1.000	—	
	95% CI Lower	1.000	—	
	Spearman’s rho	1.000 ***	—	
	*p*-value	<0.001	—	
	Kendall’s Tau B	1.000 *	—	
	*p*-value	0.014	—	
CD34_H-SCORE	Pearson’s r	0.984 ***	0.984 ***	—
	*p*-value	<0.001	<0.001	—
	95% CI Upper	0.998	0.998	—
	95% CI Lower	0.893	0.893	—
	Spearman’s rho	0.618	0.618	—
	*p*-value	0.139	0.139	—
	Kendall’s Tau B	0.548	0.548	—
	*p*-value	0.130	0.130	—
IMBV_CD34+/SMA-	Pearson’s r	0.961 ***	0.961 ***	0.967 ***
	*p*-value	<0.001	<0.001	<0.001
	95% CI Upper	0.994	0.994	0.995
	95% CI Lower	0.753	0.753	0.785
	Spearman’s rho	0.624	0.624	0.505
	*p*-value	0.135	0.135	0.248
	Kendall’s Tau B	0.562	0.562	0.410
	*p*-value	0.127	0.127	0.214

* *p* < 0.05—statistically significant. *** *p* < 0.001—strongest statistical significance.

## Data Availability

The data can be shared up on request.
